# Tight Junctions and Cancer: Targeting Claudin-1 and Claudin-4 in Thyroid Pathologies

**DOI:** 10.3390/ph17101304

**Published:** 2024-09-30

**Authors:** Jędrzej Borowczak, Dariusz Łaszczych, Katarzyna Olejnik, Jakub Michalski, Anna Gutowska, Monika Kula, Anita Bator, Marta Sekielska-Domanowska, Roman Makarewicz, Andrzej Marszałek, Łukasz Szylberg, Magdalena Bodnar

**Affiliations:** 1 Department of Clinical Oncology, Oncology Centre Prof. Franciszek Łukaszczyk Memorial Hospital, 85-796 Bydgoszcz, Poland; jedrzej.borowczak@gmail.com; 2 Department of Tumor Pathology and Pathomorphology, Oncology Centre Prof. Franciszek Łukaszczyk Memorial Hospital, 85-796 Bydgoszcz, Poland; laszczychdariusz@gmail.com (D.Ł.); l.szylberg@cm.umk.pl (Ł.S.); 3 Chair of Pathology, Dr Jan Biziel University Hospital No. 2, 85-168 Bydgoszcz, Poland; 4 Department of Obstetrics, Gynaecology and Oncology, Collegium Medicum, Nicolaus Copernicus University, 85-168 Bydgoszcz, Poland; 5 Department of Oncology and Brachytherapy, Collegium Medicum, Nicolaus Copernicus University, 85-796 Bydgoszcz, Poland; 6 Chair of Oncologic Pathology and Prophylaxis, Poznan University of Medical Sciences and Greater Poland Cancer Center, 61-866 Poznan, Poland

**Keywords:** claudin-1, claudins, thyroid cancer, prognostic marker, targeted therapy, pathology

## Abstract

**Purpose:** Claudins are tight junction proteins partaking in epithelial-mesenchymal transition and cancer progression. In this study, we investigated the expression patterns of claudin-1 and claudin-4 in thyroid pathologies, discussed their links with the pathogenesis of thyroid cancers, and reviewed the therapeutic potential of targeting claudins in cancers. **Methods:** The research group 162 cores of thyroid samples from patients (70 female and 11 male) diagnosed with thyroid adenoma, goiter, papillary, medullary, and anaplastic thyroid cancers. All samples were stained for the expression of claudin-1 and claudin-4, and the analysis of IHC was performed. **Results:** Goiter samples showed negative claudin-1 and mostly positive expression of claudin-4. Papillary thyroid cancer and thyroid adenoma showed positive expression of claudin-1, while claudin-4 was positive in papillary thyroid cancers, goiters, and adenomas. In The Cancer Genome Atlas cohort, claudin-1 and claudin-4 were overexpressed in papillary thyroid cancer compared to normal thyroid tissues. Patients with high claudin-1 expression had significantly lower 5-year overall survival than patients with low claudin-1 levels (86.75% vs. 98.65, respectively). In multivariate analysis, high claudin-1 expression (HR 7.91, CI 95% 1.79–35, *p* = 0.006) and advanced clinical stage remained statistically significant prognostic factors of poor prognosis in papillary thyroid cancer. **Conclusions:** The pattern of claudin-1 staining was pathology-specific and changed between cancers of different histology. This phenomenon may be associated with the different pathogenesis of thyroid cancers and early metastasis. The loss of claudin-1 and claudin-4 characterized more aggressive cancers. Several studies have shown the benefits of targeting claudins in cancers, but their implementation into clinical practice requires further trials.

## 1. Introduction

The epithelial layer maintains its integrity through tight junctions, desmosomes, and adherens junctions, while gap junctions enable cell communication. Junction proteins bind to the cytoskeleton, maintain the epithelial tissue’s structural integrity, regulate cell proliferation, and determine its polarity [1]. Tight junctions form a continuous strand of cell-to-cell connections on the apical-most junction, thus creating a paracellular barrier that prevents the free diffusion of solutes [2,3]. During epithelial–mesenchymal transition (EMT), a pivotal step in carcinogenesis, epithelial cells lose their capacity to form organized layers and maintain cell–cell adhesion proteins, becoming isolated and motile [4]. These changes result in the initiation of local inflammation, dysregulation of tight junction protein expression, and impaired integrity of the epithelial barrier [5,6]. EMT has also been identified as an essential step in papillary thyroid cancer (PTC) progression [7]. However, the different tight junction protein expression patterns in thyroid pathologies still need to be investigated [8].

Claudins are integral tight junction proteins that belong to the PMP22/EMP/MP20/claudin superfamily and usually contain four transmembrane domains, two extracellular loops, and one intracellular loop, with amino and carboxy-terminal sequences positioned internally [3,9]. As for now, 24 claudins have been described [3]. While claudins govern the maintenance of tight junctions throughout the life of cells, their expression and its dynamics vary between organs and tissues [10]. They regulate epithelial barrier integrity and maintain paracellular permeability. Thus, their dysregulation during carcinogenesis can have catastrophic consequences and predispose them to increased invasiveness and early metastasis [11,12,13].

The exact way claudins maintain their functions is not yet fully understood, although various models associated primarily with epithelial proliferation and differentiation have been suggested [14]. For instance, claudin-1 overexpression promoted thyroid cancer cell migration and invasion, while its knockdown reduced the viability and growth of thyroid tumors [15]. Since claudin-1 expression is induced by the protein kinase C (PKC), the up-and-downregulation of PKC activity was directly associated with claudin-1 expression in follicular thyroid cancer (FTC) cells [16]. Claudin-1 levels increased stepwise during the progression from normal thyroid tissue to thyroid cancer, but the mechanisms underlying those changes remain unknown [17]. Interestingly, claudin-1 expression correlated positively with PD-L1 levels in FTC, and PD-L1 knockdown significantly decreased claudin-1 levels and suppressed the proliferation and migration of FTC cells [18]. The dysregulation of claudin-1 expression may also be implicated in PTC tumorigenesis [19]. Exposure to ionizing radiation in the past was associated with abnormalities in claudin expression in PTC patients and the gain of chromosome band 7q11 encoding both claudin-3 and claudin-4, which may promote tumor invasiveness and metastasis [20]. PTC overexpressing claudin-1 showed a more aggressive clinical course; however, claudin-1 expression appeared to be independent of the Wnt/β-catenin pathway, through which claudin-1 increased the susceptibility to colitis-associated cancers by increasing Wnt/β-Cat^Ser552^ signaling in Notch/PI3K/Akt-dependent manner [21,22]. The loss of claudin expression is also a frequent occurrence in poorly and undifferentiated, highly aggressive thyroid cancers. The loss of cell-to-cell adhesive molecules impairs the formation of epithelial structure, facilitates the migration of cancer cells, and promotes tumor progression through the increased influx of nutrients and growth factors [23].

Claudin dysregulation was also found in other thyroid pathologies. Since claudin-1 downregulation impairs the integrality of thyrocytes within the follicle and facilitates the migration of thyroid autoantigens to the immune system, the breakage of the thyroid epithelial barrier may play a key role in the pathogenesis of HT [24,25]. Compared to normal thyroid epithelium, thyroid epithelial cells affected by autoimmune Hashimoto thyroiditis (HT) showed reduced claudin-1 levels. On the other hand, claudin-1 expression was increased in the follicular epithelial dysplasia (FED) area within HT tissue. Since FED is considered a precursor of papillary cancer, upregulation of claudin-1 may be associated with neoplastic changes [17]. Considering the role of claudin-1 in thyroid premalignancy-to-malignancy transition, it has also been demonstrated that expression of claudin-1 increases in solitary dominant nodules (SDN) compared to normal gland tissue [26]. According to the latest meta-analysis, SDN indicated a higher risk of thyroid cancer occurrence compared to multinodular goiter [27]. Contemporary literature suggests that the dysregulation of claudin-1 may play an important role in the pathogenesis of autoimmune disorders of the thyroid gland, and claudin-1 may be a potential linkage between chronic inflammation and thyroid tumorigenesis. Therefore, evaluating claudin-1 expression may be an important diagnostic marker when examining thyroid lesions.

This study explored the concept of targeting claudin-1 and claudin-4 in thyroid cancer. First, we analyzed the expression patterns of claudin-1 and claudin-4 in 162 samples with diagnosed thyroid pathologies. Then, we discussed the association between our results, reports from the literature, and the pathogenesis of thyroid cancers. Finally, we reviewed the emerging claudin-oriented therapies that provide a unique insight into thyroid cancer treatment.

## 2. Results

### 2.1. Basic Characteristics of the Research Group

The research cohort comprised 162 thyroid tissue samples collected from 81 patients after total or subtotal thyroidectomy. One hundred forty samples were obtained from female and 22 from male patients. The mean patients’ age was 50.21 years. Sixty-two goiter samples, fifty-eight adenoma samples, and forty-two cancer samples were examined. In the cancer group, we found 22 papillary thyroid cancers (PTC), 10 medullary thyroid cancers (MTC), and 10 anaplastic thyroid cancers (ATC). Among them were 10 pT1-stage cancers, 8 pT2-stage cancers, 4 pT3-stage cancers, and 6 pT4-stage cancers (Table 1). All normal thyroid tissue samples were used as a control and showed positive claudin-1 and claudin-4 expressions.

### 2.2. Staining Patterns of Claudin-1 and Claudin-4

During the initial histopathological examination, we analyzed the staining patterns of gathered specimens (Table 2). Positive claudin-1 expression was observed in 31% of adenomas (18/58) and 39% of cancers (16/42). Only papillary thyroid cancers showed claudin-1 staining (16/22). No goiter, medullary thyroid cancer, or anaplastic thyroid cancer expressed claudin-1.

Immunostaining for claudin-4 was positive in 97% of goiters (60/62), 72% of adenomas (42/58), 91% of papillary carcinomas (20/22), and 20% of anaplastic carcinomas (2/10). All medullary carcinoma samples were negative for claudin-4 expression.

Claudin-1 staining patterns differed depending on the type of thyroid pathology and changed between cancers of different origins. Positive expression of claudin-4 was more prevalent than positive expression of claudin-1 in almost all types of samples, but its expression did not differ between thyroid pathologies of a different type.

### 2.3. Claudin-1 and Claudin-4 Expression in Thyroid Pathologies

The expressions of claudin-1 and claudin-4 were analyzed in all thyroid pathologies. In goiter, adenoma, medullary cancer, and anaplastic cancer tissues, the median expression of claudin-1 was 0. In papillary carcinoma samples, the expression was higher than in other pathologies and reached 17.65 (*p* < 0.05).

Claudin-4 expression was the highest in papillary thyroid carcinoma tissues and was significantly higher than in adenomas and goiters (74.33 vs. 51.87 vs. 47.22, respectively; *p* < 0.05). There was no statistical difference between the median expression of claudin-4 in goiters and adenomas. The median expression of claudin-4 in MTC and ATC samples was 0.

**Figure 1:**
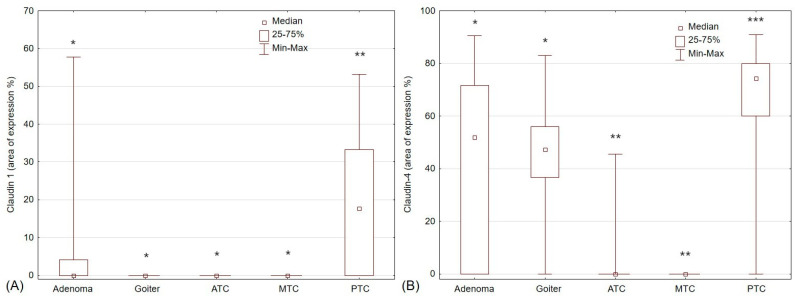
The expression of (**A**) claudin-1 in thyroid pathologies (*p* < 0.0001); (**B**) claudin-4 in thyroid pathologies (*p* < 0.0001). PTC—papillary thyroid cancer; MTC—medullary thyroid cancer; ATC—anaplastic thyroid cancer; —*, **, and *** indicate differences in median expressions, with groups marked with *, **, and *** being statistically different from each other (*p* < 0.05).

Papillary thyroid cancer was the only thyroid pathology with significant claudin-1 expression. Goiters, MTCs, and ATCs did not express claudin-1. Claudin-4 expression was positive in PTCs, goiters, and adenomas but negative in medullary and anaplastic thyroid cancers (Figure 1).

### 2.4. The Analysis of TCGA Papillary Thyroid Cancer Cohort

Considering that only PTC expressed claudin-1 in our cohort, we decided to investigate the associations between claudin-1 and claudin-4 expression and clinicopathological features of PTC in a group of 501 patients extracted from The Cancer Genome Atlas database [28]. The basic patient characteristics are summarized in Table 3. The mean age of patients was 47.4 years (range: 15–89 years), and the median follow-up was 2.57 years.

**Figure 2:**
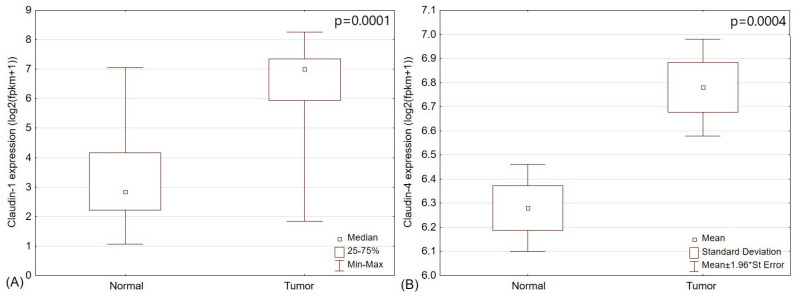
Papillary thyroid cancer showed higher expression of (**A**) claudin-1 and (**B**) claudin-4 compared to matched normal thyroid tissue samples (*p* = 0.0001 and *p* = 0.0004, respectively).

First, we compared the expression of claudin-1 and claudin-4 between PCT and matched normal thyroid tissue samples (*n* = 52). Both markers were overexpressed in PTC compared to normal tissues (*p* = 0.0001 and *p* = 0.0004, respectively) (Figure 2).

Claudin-1 expression was significantly higher in patients with lymph node involvement than in patients without nodal disease, and claudin-1 was significantly lower in patients with stage II disease than in other patients (*p* < 0.05). Patients who were 45 or younger had lower claudin-1 than older patients (*p* = 0.01). We found no association between claudin-1 expression and patients’ sex, clinical stage, and distant metastasis (*p* > 0.05). 255 (60.9%) tumors had low claudin-1 expression, and 246 (49.1%) tumors had high claudin-1 expression. Patients in the low-claudin-1 group had significantly lower 5-year overall survival than patients with high-claudin-1 tumors (86.75% vs. 98.65%; *p* = 0.001) (Figure 2). Low claudin-1 was an independent prognosis factor of shorter patients’ survival (HR = 8.06 [CI 1.8–35.6]; *p* = 0.0059).

**Figure 3:**
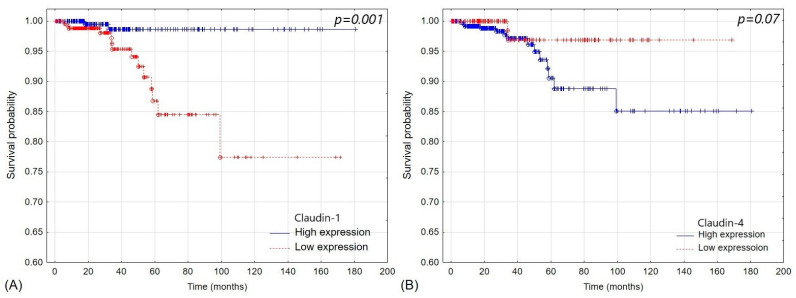
Survival differences in groups of low and high expression of (**A**) claudin-1 (*p* = 0.001) and (**B**) claudin-4 (*p* = 0.07).

Claudin-4 expression was higher in patients without nodal disease than in patients with lymph node involvement (*p* < 0.05) but did not correlate with other clinicopathological features of PTC (*p* > 0.05). Claudin-4 levels were significantly lower in patients with stage II and IV disease than in stage I and III (*p* < 0.05). On the other hand, three hundred sixty-five (72.85%) tumors had high expression claudin-4 expression, and 136 (27.15%) tumors had low claudin-4 expression. There was no significant difference in survival between patients with high and low claudin-4 expression (*p* = 0.07) (Figure 3).

In univariate analysis, low expression of claudin-1 and higher clinical stage were prognostic of shorter patient survival (*p* < 0.05). In multivariate analysis, higher clinical stage (HR 3.38, CI95% 0.17–9.77, *p* = 0.025) and low claudin-1 expression (HR 7.91, CI95% 1.79–35, *p* = 0.006) retained significance as independent prognostic factors of shorter survival in papillary thyroid cancer (Table 4).

## 3. Discussion

### 3.1. The Role of Claudins in Thyroid Pathologies

Claudins’ dysregulation has been recently associated with tumor progression and metastases; hence, claudins’ overexpression has become a potential therapeutic target [15,29]. Despite previous studies, the patterns of claudin expression in thyroid pathology remain unclear [5,8,30]. This article aimed to analyze the expression of claudin-1 and claudin-4 in various thyroid tumors. We found that claudin expression differs between thyroid pathologies and may be associated with patient prognosis. Goiter and adenoma samples showed low or no claudin-1 expression, suggesting the deregulation of the tight junction barrier. The levels of claudin-1 varied significantly between thyroid cancers—claudin-1 was overexpressed in PTC, but MTC and ACT did not express claudin-1. The situation differed slightly regarding claudin-4, expressed by goiters, adenomas, and PTC but not by MTC and ATC. In view of potential claudin-targeted therapies in thyroid pathologies, our findings need to be discussed in a broader clinical context.

Disruptions in claudin signaling may diminish the functionality of the tight junction barrier [11]. Losing tight junctions enables cancers to invade blood vessels and metastasize. This phenomenon is more prevalent in undifferentiated cancers, but surprisingly, the upregulation of tight junction proteins can also be found in the early stages of carcinogenesis [31]. In our study, claudin-4 was expressed in well-differentiated samples, both benign (goiter, adenoma) and malignant (papillary carcinoma). Still, Süren et al. found no difference between the expression of claudin-4 in benign and malignant tumors. Their expression was also similar between dominant nodules of multinodular goiter, follicular adenoma, and follicular carcinoma (*p* > 0.05) [32]. In contrast, claudin-1 was expressed more frequently in malignancies than benign pathologies (*p* < 0.001). In our cohort, claudin-1 was positive in papillary carcinoma and, to a smaller degree, in adenomas (Table 2 and Table 3). Epithelial cell-derived cancer, such as papillary carcinoma, follicular carcinoma, and anaplastic thyroid carcinoma, could be expected to have high expression of claudins [33]. However, the lack of claudins indicates disease progression since ATC arises from pre-existing, better-differentiated malignancy. Claudin knockdown likely occurred during the epithelial–mesenchymal transition and corresponds to the high metastatic potential of ATC [7].

In our cohort, claudin-1 and claudin-4 expression patterns were similar, with nearly uniform positive expression in papillary carcinomas, negative expression in undifferentiated and medullary carcinomas, and varied expression in adenomas. Only goiters showed negative claudin-1 and positive claudin-4 staining, similar to those reported in normal thyroid tissues [23,34]. Claudin-1 and claudin-4 expressions were negative in medullary carcinoma and most anaphylactic carcinoma samples, indicating tight junction loss in aggressive thyroid cancers, suggesting that the downregulation of claudins is associated with the de-differentiation of thyroid cancer [23]. Furthermore, claudin-1 was overexpressed in papillary thyroid cancer, while claudins 1, 4, and 7 were underexpressed in undifferentiated thyroid carcinomas. While those results align with ours, the authors reported that claudin-4 was expressed in ~40% of medullary carcinomas. In our cohort, all MTCs were negative for claudin-4 expression [21]. Papillary carcinoma exhibited high expression of both claudin-1 and 4, and claudin-1 expression was higher in patients with nodal disease than in patients without nodal disease. In contrast, high claudin-1 expression correlated with longer patient survival (Figure 3); the diagnostic value of claudin-1 is debatable due to its unpredictable staining patterns [21,35]. This constituted a significant challenge in adopting claudin-targeted therapies for clinical use. The loss of epithelial claudin-1 disrupts cell polarity and integrity. However, high levels of claudin-1 were associated with increased aggressiveness of follicular thyroid carcinoma, implying that its role may be tissue-specific [16]. In in vivo studies, claudins showed varied degrees of tumor-suppressive properties, inhibiting tumor cell proliferation, migration, and invasion [36]. Claudin-4 has also been recently considered a potential marker for targeted therapy and chemotherapy in ovarian and pancreatic cancers [37,38]. Nevertheless, this strategy is not devoid of flaws, especially when brain metastases are concerned [39].

### 3.2. Targeting Claudin 1 and Claudin 4 in Thyroid Cancer

**Figure 4:**
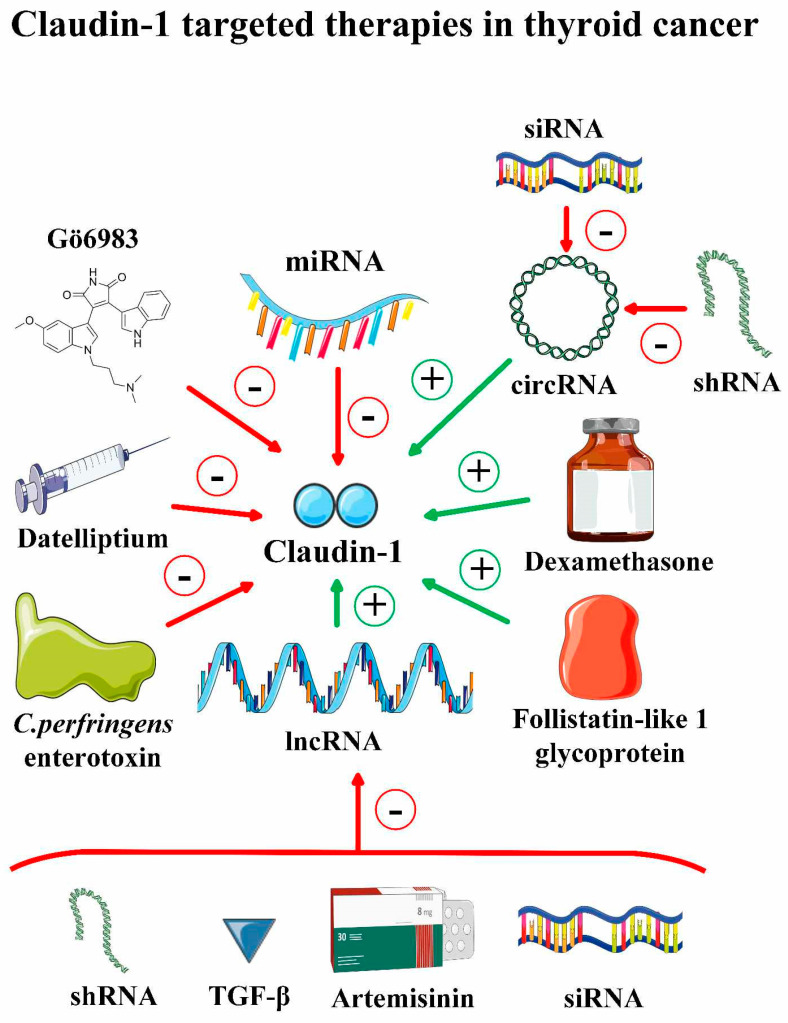
Therapeutic strategies targeting claudin-1 in thyroid cancer. Downregulation of claudin-1 expression may be achieved using several agents, including PKC inhibitor Gö6983, RET inhibitor datelliptium, and *C. perfringens* enterotoxin. In addition, exogenic delivery of anti-tumor microRNA using non-viral and viral-vector methods is another promising strategy. The anti-cancer effect may also be achieved indirectly through targeting pro-tumor factors, including circular RNA or long non-coding RNA, with several methods, including RNA interference or antimalarial artemisinin. A therapeutic approach aiming to upregulate claudin-1 expression with the use of dexamethasone or follistatin-like one glycoprotein may inhibit cell migration and have an anti-cancer effect in the case of anaplastic or highly-differentiated metastatic thyroid cancer.

In the scope of the emerging evidence, claudin loss appears to be a major step in cancer progression. The loss of epithelial barrier integrity facilitates cancer entry into circulation and contributes to early metastasis. Available preclinical studies indicate that targeting claudins, especially claudin-1, may be a promising therapeutic approach in thyroid cancer (Figure 4). Decreasing claudin expression could enhance drug penetration into the tumor, potentially improving the effectiveness of therapy. On the other hand, restoring the epithelial barrier integrity may reduce the risk of early metastasis and improve patient prognosis [40]. However, their efficacy and safety still need to be elucidated. We reviewed the literature and summarized the previous reports to highlight the potential role of claudin-1 and claudin-4 targeted therapy in thyroid cancer (Table 5).

Alterations in the RET gene are key drivers of papillary and medullary thyroid carcinoma tumorigenesis. While claudin-4 expression varies between MTC specimens, datelliptium, an inhibitor of Rearranged during Transfection (RET) proto-oncogene, downregulated mesenchymal markers and claudin-1 in MTC cells and suppressed EMT, exhibiting a significant anti-cancer effect in vivo (xenograft model) through RET inhibition [41]. The FDA has recently approved two RET inhibitors, selpercatinib and pralsetinib, for treating advanced or metastatic thyroid cancer with a RET gene fusion or mutation [42]. Dexamethasone, a synthetic glucocorticoid, caused an anti-metastasis effect in FTC-133 cells, possibly through the upregulation of tight junction proteins, including claudin-1 and zonula occludens 1 (ZO-1). It seems that dexamethasone, through the restoration of various adhesion molecules, may participate in the re-dedifferentiation of highly metastatic thyroid cancer [43].

MicroRNA (miRNA) emerged as a potential therapeutic modality in thyroid cancer. miRNAs are a subtype of non-coding RNA involved in cell-to-cell communication. Due to their ability to regulate the expression of various genes, miRNAs may act either as tumor suppressors or oncogenes. miRNA-331-5p regulates the critical functions of thyroid cancer cells, including motility and survival [44]. miRNA may also regulate the expression of tight junction proteins, including claudin-1, in thyroid malignancies. IHH-4 and TPC-1 papillary thyroid cancer cells transfected with miRNA-129-5p had significantly downregulated expression of claudin-1 and caused cancer cells apoptosis [45]. Furthermore, mRNA-129-5p expression is regulated by circular RNA (circRNA) circ_0027446, and the expressions of both miRNAs were adversary correlated in papillary thyroid cancer, suggesting that claudin-1 overexpression in PTC is independent of circ_0027446 [45]. Despite that, targeting circ_0027446 or delivering tumor-suppressing miRNA-129-5p to cancer cells may be a potential anti-claudin-1 therapy in thyroid cancer. Unfortunately, studies targeting circ_0027446 in thyroid cancer are lacking, while off-target toxicity, lack of tissue/cell specificity, or immunogenicity remain significant challenges in targeting circRNA [46]. While reports from other cancers suggest their efficacy, further trials are needed [47].

The combination of tumor-suppressing miRNA and other anti-cancer drugs may also improve the efficacy of therapy [48]. For instance, the transfection of miRNA-199a-5p mimics suppressed PD-L1 and claudin-1 expression in FTC-133 cells [18]. Combining miRNA-199-5p with anti-PD-L1 agents improves anti-cancer therapy’s efficacy by downregulating claudin-1. While the role of such combined approaches in thyroid cancer remains unexplored, atezolizumab (an anti-PD-L1 agent) increased the expressions of claudin-1 and occludin in murine ileum during sepsis [49]. Currently, there is an ongoing clinical trial that evaluated the efficacy and safety of ALE.C04 in monotherapy (first-in-class anti-claudin-1 monoclonal antibody) and combination with pembrolizumab (anti-PD-L1) in head and neck cancer (NCT06054477). Further trials are needed to fully uncover the association between miRNA-199-5p and anti-PD-L1 in thyroid cancer treatment.

mi9RNA-101-3p is another microRNA that suppresses PTC cell migration and invasion by downregulating the miRNA-101-3p/Claudin-1 axis. Mechanistically, long non-coding RNA X-inactive specific transcript (lncRNA XIST) bound to and downregulated miRNA-101-3p, promoting cancer cell migration and increasing claudin-1 expression [50]. The miRNA-101-3p expression may also be restored by targeting circular RNA circRTN1 [51]. Targeting lncRNA XIST may also be a promising approach to treating thyroid cancer. Artemisinin, an antimalarial drug, suppresses the pro-oncogenic effect of XIST and inhibits aerobic glycolysis, proliferation, and metastatic potential of thyroid cancer cells through the degradation of hypoxia-inducible factor 1α (HIF-1α) [52]. To date, lncRNA XIST has been targeted in thyroid cancer using, e.g., siRNA, short hairpin RNA (shRNA), and TGF-β [50,53,54,55,56].

While data regarding thyroid cancer are scarce, the association between miRNA and claudin-1 has been demonstrated in other thyroid pathologies, including Hashimoto’s thyroiditis (HT). For instance, in HT tissue samples, thyrocytes transfected with miRNA-142-5p-loaded lentiviral vector had significantly downregulated expression of claudin-1, and areas with high miRNA-142-5p levels lacked claudin-1 expression [24]. Therefore, restoring the expression of tumor-suppressing miRNA may be a potential therapeutic approach in thyroid cancer. The reports from preclinical studies support the need for further studies evaluating the efficacy and safety of that potential anti-cancer treatment, but further investigations are needed.

Most studies indicate that claudin-1 overexpression exhibits pro-oncogenic activity in papillary thyroid cancer. However, its role in other thyroid pathologies remains unknown. For example, secretory extracellular glycoprotein follistatin-like 1 (FSTL1) upregulated claudin-1 and claudin-4 genes, increasing the expression of epithelial gene markers and reducing the metastatic potential of 8505C anaplastic thyroid cells [57].

Targeting claudin-1 and claudin-4 is a promising approach to managing thyroid cancers. Unfortunately, the available literature is limited, and more clinical trials are needed to evaluate the efficacy and safety of claudin-1/4-targeted therapies in thyroid malignancies.

**Table 5:** Summary of preclinical studies targeting claudin-1 in various malignancies (up and downregulation) published between 2019 and 2024.

Agent/Intervention	Settings	Biological Effects	Impact on Claudin-1	Ref.
PMTPV (short peptide)	Lung adenocarcinoma; A549 cell line	-PMTPV increases the chemosensitivity to doxorubicin	-PMTPV selectively decreased claudin-1 protein level by promoting its endocytosis and degradation	[58]
Tranilast and zoledronic acid (EMT inhibitors)	Small cell lung cancer; SBC-3 cell line	-Co-treatment with both agents increased the chemosensitivity of claudin-1-overexpressing SBC-3 cancer cells to doxorubicin	N/A	[59]
SCLC-derived exosomal miR-375-3p inhibitor (anti-miR-375)	Small cell lung cancer; cell lines and murine xenograft	-Decreased permeability of the vascular barrier and SCLC metastasis in vivo	-Reversed claudin-1 downregulation in endothelium	[60]
Claudin-1 specific siRNA	Head and neck squamous cell carcinoma; FaDu and SNU1041 cell lines; murine xenograft	-Inhibition of tumor progression through the activation of the AMPK signaling pathway -Suppression of cancer cell proliferation and migration -Negative regulation of the EMT	-Claudin-1 downregulation	[61]
Claudin-1 specific monoclonal antibody (mAb)	Hepatocellular carcinoma; cell lines and murine xenograft	-Anti-cancer effect on HCC cells, including sorafenib-and nivolumab-resistant cells; suppression of HCC growth in vivo through the promotion of apoptosis -Promotion of tumor metabolism reprogramming -Potential enhancement of the antitumor immunity	-Targeting claudin-1	[62]
Claudin-1-RNA interference lentivirus (LV-CLDN1-RNAi)	Gallbladder cancer; SGC996 and GBC-SD cell lines	-Significant decrease in the proportion of cancer cells in the S phase of the cell cycle -Promotion of apoptosis and inhibition of the invasion capacity of cancer cells	-Claudin-1 downregulation	[63]
siRNA targeting GALNT5 (siGALNT5) expression	Cholangiocarcinoma; KKU-213 and KKU-214 cell lines	-Suppression of the growth, migration, and invasion of ChCC cells -Suppression of GALNT5 may inhibit the EMT in ChCC through upregulation of claudin-1	-siGALNT5 increased claudin-1 expression -GALNT5 decreased the expression of claudin-1 through the activation of Akt and Erk signaling	[64]
Lycopene	Cutaneous squamous cell carcinoma; COLO-16 cell line	N/A	-Downregulation of claudin-1 expression through activation of ERK, JKN, and MTORC1 pathways and inhibition of autophagy in cancer cells	[65]
MIGR1 plasmid expressing p63 isotype ΔNp63α (MIGR1-ΔNp63α)	Cervical squamous cell carcinoma; SiHa cell line	-Suppression of tumor growth in vivo -Increase in K5 and involucrin expression in CSCC cells -Promotion of epithelial differentiation -Inhibition of angiogenesis -Reduction of cisplatin resistance in CSCC	-Upregulation of claudin-1 expression both in vitro and in vivo -Claudin-1 correlates positively with ΔNp63α in CSCC tissues	[66]
pMIGR1 plasmid expressing claudin-1 (pMIGR1-CLDN1)	Cervical squamous cell carcinoma; SiHa cell line and murine xenograft	-Claudin-1 overexpression suppressed cancer cell proliferation and migration and promoted their apoptosis -Claudin-1 promoted cell cycle arrest and inhibited CSCC growth in vivo	-Overexpression of claudin-1 in CSCC cells	[67]
YM201636 (PIKfyve inhibitor)	Ovarian cancer; HEYA8 and DOV13 cell lines	-Disruption of spheroid formation, inhibition of cell proliferation and migration -YM201636 may limit ovarian cancer spread and relapse	-Inhibition of claudin-1 recycling to the cellular membrane	[68]
Withaferin A	Oral squamous cell carcinoma; Ca9.22, HSC-4 and HN22 cell lines; murine xenograft	-Impair of cancer cells’ motility via downregulation of claudin-1 -Suppression of OSCC distant metastasis in vivo (murine model) with no systemic toxicity	-Claudin-1 downregulation	[69]
3,3′-Diindolylmethane (DIM)	Esophageal squamous cell carcinoma; TE1 and KYSE150 cell lines; murine xenograft	-Suppression of the EMT process through the upregulation of claudin-1 in vivo -The anti-EMT effect of DIM is associated with the modulation of AHR through repression of the COX2/PGE2 pathway	- Upregulation of claudin-1 in vivo	[70]
MiR-27b-3p expressing pcDNA3.1 vector	Esophageal squamous cell carcinoma; TE-1 and EC9706 cell lines	-Suppression of proliferation and migration in EC9706 cells -Decrease of Nrf2 expression; -Potential inhibition of EMT through targeting Nrf2	- Downregulation of claudin-1	[71]
Resveratrol	Gastric cancer; SGC7901, GES-1, MGC803 and AGS cell lines	-Inhibition of the proliferation, migration, and invasion together with the promotion of apoptosis of gastric cancer cells through downregulation of miR-155-5p -Downregulation of c-Myc, cyclin D1, and Bcl-2 and upregulation of caspase-3 expression	-Downregulation of claudin-1	[72]
European Olive (*Olea* *europaea L.*) leaf extract (OLE)	Gastric cancer; AGS cell line	-Sensitization of gastric cancer to chemotherapy by targeting claudin-1 -Inhibition of the EMT process and suppression of AGS cell migration and stem-like phenotype	-Downregulation of claudin-1 in AGS cells; more prominent in cells treated with OLE+5-FU and OLE+Cisplatinum	[73]
MiR-633 lentivirus vector (LV-miR-633)	Gastric cancer; SGC-7901 and HGC-27 cell lines	-Inhibition of cancer cell proliferation, induction of cell cycle arrest at G0/G1, and promotion of cancer cell apoptosis -Suppression of cancer cell migration and invasion -MiR-633 exhibits anti-cancer effects through targeting MAPK1	-MiR-633 may target the claudin-1 gene and negatively regulate its expression	[74]
shRNA targeting Ephrin-A2	Prostate cancer; LNCaP, PC-3, and DU145 cell lines; murine xenograft	-Suppression of tumor growth, metastasis to lymph nodes and lungs, and angiogenesis in vivo -Targeting ephrin-A2 may decrease prostate cancer metastasis by targeting EMT-related markers	-Upregulation of claudin-1	[75]
Glutamine (Gln) deprivation, Diazo-O-norleucine ( glutaminase inhibitor) (DON)	Breast cancer; MDA-MB-231 and MCF-7 cell lines	-Gln-deprivation and DON treatment induced epithelial differentiation of breast cancer stem cells and reduced their stemness -Targeting glutamine may decrease breast cancer invasion and metastasis	-Upregulation of claudin-1	[76]
Brahma (BRM) overexpression through transient transfection	Breast cancer; MCF-7 and MDA-231 cell lines	-Suppression of migration and invasion of cancer cells -TGF-β treatment disrupted the BRM-induced upregulation of claudin1/4	-Upregulation of claudin-1 and claudin-4 through acetylation of histones surrounding the claudin1/4 promoters	[77]
C150 (2-[2-(5-nitro-2-thienyl)vinyl]quinoline)	Pancreatic ductal adenocarcinoma; PANC-1 cell line; murine xenograft	-Reduction of tumor growth in vivo -C150 treatment is associated with a statistically significant loss of mean body weight	-Upregulation of claudin-1 and ZO-1 in vivo through the promotion of Snail proteasomal degradation	[78]
miRNA-193b-5p mimic transfection	Pancreatic ductal adenocarcinoma; PANC-1 cell line and murine xenograft	-Suppression of cancer cell proliferation, migration, invasion, and EMT process through targeting eEF2K signaling pathways -Lipid-nanoparticles-based delivery of miR-193b in vivo significantly reduces tumor growth and eEF2K expression	-Upregulation of claudin-1 and E-cadherin	[79]
N-(phenylcarbamothioyl)-2-napthamides as claudin-1 inhibitors	Colorectal carcinoma; SW620 cell line	-Compound VM-A-155B is a promising anti-CRC claudin-1 inhibitor (best activity, in vitro and in vivo properties)	-Targeting claudin-1 in CRC cells	[80]
*Lactobacillus plantarum*— derived metabolites (LDMs)	Colorectal carcinoma; HCT-116 and HCT-116/5FUR cell lines	-LDMs enhance the sensitivity and cytotoxicity of 5-FU in resistant cancer cells -LDMs inhibit the metastatic potential of resistant cancer cells -LDMs’ anti-cancer activity is associated with the downregulation of claudin-1	-LDMs alone and in combination with 5-FU downregulate the claudin-1 in HCT-116/5FUR	[81]
tRNA-derived fragment tRF-20-M0NK5Y93	Colorectal carcinoma; RKO and SW480 cell lines	-Inhibition of CRC cell migration, invasion, and metastatic properties through inhibition of claudin-1 -Hypoxic conditions downregulate tRF-20-M0NK5Y93 expression	- Direct and negative regulation of claudin-1 expression	[82]
PDS0330, a first-generation inhibitor of claudin-1	Colorectal carcinoma; HCT116, SW480^cld1^, and SW620 cell lines; in silico; murine xenograft	-PDS-0330 is characterized by favorable pharmacodynamics and pharmacokinetics in vitro and in vivo -PDS-0330 has higher affinity and specificity binding to claudin-1 compared to other claudins -Reduction in anoikis and therapy resistance of cancer cells -Inhibition of tumor growth in vivo with no major cytotoxicity -Synergistic effect with 5-FU in vivo -Reduction of claudin-1-dependent CRC chemoresistance	-Inhibition of claudin1-mediated signaling through interferential with claudin-1-Src binding	[83]
Oxaliplatin and anti-CLDN1 antibody-drug conjugate (6F6-ADC)	Colorectal carcinoma; SW620 and HCT116 cell lines; murine xenograft	-Oxaliplatin-induced claudin-1 expression is associated with p38/GSK3β/Wnt-β-catenin pathway -Oxaliplatin resistance is mediated by claudin-1-induced apoptosis resistance -6F6-ADC suppresses CRC growth in vivo -Sequential oxaliplatin +6F6-ADC exhibits a stronger anti-cancer effect and increased survival than oxaliplatin monotherapy	-Standard CRC chemotherapy upregulates the claudin-1 expression in CRC in vitro and in vivo -6F6-ADC targets extracellular part of claudin-1 in CRC	[84]

### 3.3. Targeting Claudin-1 and Claudin-4 in Other Malignancies

The therapeutic strategies targeting claudin-1/4 are intensively investigated in experimental oncology (Figure 4). To highlight the potential role of claudin-1 and claudin-4 targeted therapies in thyroid cancer, we revised and summarized relevant studies published between 2019 and 2024 (Table 6). Among them, the potential chemotherapy-enhancing activity of claudin-targeting agents has gained particular attention. The mechanism underlying this phenomenon is associated with the dysregulation of tight junction integrity, which facilitates anticancer agent penetration deep into tumor tissue [58,59,73,81,83,84,85,86,87]. Claudin-targeted therapy may also enhance the efficiency of immunotherapy through immunomodulation of TME [62,85]. These properties are currently being investigated in HNSCC (NCT06054477). Anit-claudin treatment may suppress the EMT, facilitating tumor spread by promoting epithelial marker expression. On the contrary, claudin-1/4 upregulation may suppress tumor migration, invasion, and EMT by affecting several signaling pathways [61,69,71,73,81,82,88,89]. To fully elucidate the role of claudin-1/4 in EMT and tumor metastasis, further trials are needed. The inhibition of claudin-4 may affect DNA damage repair in cancer cells and enhance the effectiveness of PARP inhibitors in ovarian cancer [90]. Overexpression of claudins can potentially be targeted by cancer cell-specific therapies, including systems delivering nanoparticles or liposomes. Due to its high cancer specificity, this strategy may increase the effectiveness of systemic treatment without additional toxicity [91,92].

**Table 6:** Summary of preclinical studies targeting claudin-4 in various malignancies published between 2019 and 2024.

Agent/Intervention	Malignancy	Biological Effects	Impact on Claudin-4	Ref.
*Clostridium perfringens* enterotoxin Obstructing Protein (COP-1), synthetic antibody fragment (sFab)	Structural, biophysical, and biochemical analysis with the use of single-particle cryogenic electron microscopy (cryo-EM)	-COP-1 is a monovalent and stable molecule and binds with high affinity and selectivity to claudin-4	-COP-1 binds with the extracellular surface of human claudin-4	[93]
siRNA targeting PVT1 exon 9 expression (siPVT1e9)	Triple-negative breast cancer; MDA MB 231 cell line	-Suppression of migratory capacity of MDA MB 231 cancer cells	-Knockdown of PVT1 exon 9 results in re-expression of claudin-4 protein with no impact on claudin-4 mRNA level	[88]
Fusion toxin secreted by *Bifidobacterium longum* (*B. longum*-C-CPE-PE23)	Breast cancer; MCF-7, MDA-MB-468 and HCC1937 cell lines; murine xenograft	-Suppression of tumor growth in vivo -Treatment did not affect body weight, ALT, AST, and BUN levels	-C-CPE-PE23 is cytotoxic to cells expressing claudin-4	[94]
Anti-claudin-4 extracellular domain antibody (4D3)	Breast cancer; MCF-7 and MDA-468 cell lines; murine xenograft	-4D3 enhances paclitaxel-induced anti-cancer effect in vitro -4D3 facilities paclitaxel intracellular penetration -4DC exhibits immunomodulatory effect on TME in breast cancer -Three-way combination of paclitaxel, tamoxifen, and 4D3 significantly prolonged survival of mice -Combined treatment of paclitaxel, zoledronic acid, and 4D3 reduced the growth of breast cancer bone metastasis	-Targeting cells expressing claudin-4	[85]
Claudin-4 targeting short hairpin RNA (shCLDN4); claudin-4 expressing pLenti-C-mGFP vector (CLDN4-GFP); claudin-4-disrupting peptide (CMP)	Ovarian cancer; OVCAR3, PEO4, OVCAR8, and OVCAR4 cell lines	-Claudin-4 overexpression in ovarian cancer drives paclitaxel resistance -Claudin-4 expressing cells exhibited a higher response to paclitaxel, but no cisplatin, after cotreatment with CMP or shCLDN4 -Cancer cells lacking claudin-4 are characterized by inhibition of G2-M progression of cell cycle and abundant of mitotic figures -Claudin-4 regulates the polymerization of microtubules, which may be a potential paclitaxel-resistance mechanism	-Claudin-4 knockdown after shCLDN4 -Claudin-4 overexpression in cells transfected with CLDN4-GFP -CMP inhibits claudin-4 activity	[95]
Claudin-4 short hairpin RNA lentiviral vector (shCLDN4); CMP	Ovarian cancer; in vitro studies	-Claudin-4 expression correlates with DNA damage repair -Claudin-4 KO increases sensitivity to PARP inhibitors by affecting nonhomologous end joining DNA repair -Olaparib/CMP cotreatment inhibited proliferation regardless of the claudin-4 expression in tumor	-Claudin-4 knockdown after shCLDN4 -CMP inhibits claudin-4 activity	[90]
Celastrol	Gastric cancer; SGC7901 and BGC823 cell lines	-Celastrol inhibits proliferation, migration, and invasion of cancer cells by targeting the FOXA1/CLDN4 axis and later inhibition of PI3K/AKT signaling	-Celastrol targets FOXA1 which results in claudin-4 downregulation	[89]
Anti-CLDN4 antibody; 4D3	Gastric cancer; TMK-1 and MKN74 cell lines; murine xenograft	-4D3 treatment downregulates claudin-4, EGFR, and VEGF in cancer cells and inhibits cell proliferation -4D3 decreases gastric cancer stemness markers -Combined 4D3 and cis-diamminedichloroplatinum (CDDP) have a synergic anti-cancer effect -4D3 increases CDDP intracellular concentration -4D3 monotherapy inhibited the growth of well-differentiated gastric cancer in vivo but not poorly differentiated one -Concurrent CDDP and 4D3 exhibit higher anti-cancer activity compared to monotherapy in both tumors and improve mice survival	-4D3 decreases claudin-4 expression	[86]
4D3	Pancreatic ductal adenocarcinoma; MIA-PaCa-2 cell line; murine xenograft	-4D3 decreased HIF-1α expression in PDAC cells -4D3 increased intracellular concentration of 5-FU -Simultaneous treatment of 4D3 increased the antitumoral effect of the FFX regimen, i.e., oxaliplatin (L-OHP), irinotecan (CPT-11), and 5-FU -4D3 and FFX suppressed tumor growth in vivo and prolonged mice survival -A half dose of FFX is a significantly safer approach than a full dose of FFX with a similar anti-cancer effect	-4D3 inhibits claudin-4	[87]
Doxorubicin-loaded- *Clostridium perfringens* enterotoxin peptide-conjugated polysialic acid nanoparticles (DOX-C-SNPs)	Pancreatic ductal adenocarcinoma; KPC960 murine PDAC cell lines; murine allograft	-DOX-C-SNPs release specifically and effectively doxorubicin in cancer cells -DOX-C-SNPs provide low non-specific/off-target uptake of doxorubicin and high stability -DOX-C-SNPs exhibited a higher anti-cancer effect compared to DOX-SNP and free doxorubicin in vivo -DOX-C-SNPs were associated with reduced doxorubicin-related systemic toxicity and prolonged mice survival	-DOX-C-SNPs bind with the extracellular domain of claudin-4 expressed on the surface of cancer cells	[91]
Doxorubicin (Dox)-loaded, CPE17-conjugated liposomes (D@C-LPs)	Pancreatic ductal adenocarcinoma; ASPC-1 and KPC960 cell lines; murine allograft and xenograft	-D@C-LPs bind specifically with claudin-4 expressing cancer cells -Claudin-4 in case of PDAC is exposed superficially, whereas access to claudin-4 in case of normal pancreatic tissue is hindered -D@C-LP resulted in significant suppression of tumor growth in vivo and prolonged mice survival compared to free doxorubicin -D@C-LP was associated with no major systemic toxicities	-D@C-LPs targets claudin-4 positive cancer cells	[92]
Y364947, TGFβ1 inhibitor	Colorectal cancer; HT29 and HCT116 cell lines	-LY364947 suppressed claudin-4 mRNA and protein levels in both CRC cell lines	-Y364947 downregulates claudin-4 expression through TGFβ1 signaling inhibition	[96]

### 3.4. Limitations

The presented study has several limitations that need to be acknowledged while drawing conclusions. We examined a total of 162 samples, they were collected from 81 resection specimens. This approach allowed us to assess the heterogeneity of thyroid pathologies and compare claudin expression in different areas of the examined pathology. While the achieved results were statistically significant, a follow-up analysis of a larger research cohort is necessary. Furthermore, the methodology used, including digital-assisted tools for IHC assessment, may differ slightly from the traditional manual assessment and should be taken into account when compared with other reports. Furthermore, since IHC is a semi-quantitative method, claudin-negativity corresponded to a total lack of claudin expression; however, it remains to be investigated whether the protein in claudin-protein cells was fully functional.

## 4. Materials and Methods

### 4.1. Patients and Tissue Samples

All tissue specimens were collected from patients diagnosed with thyroid adenoma, thyroid cancer, or goiter between January 2012 and December 2013. The specimen was classified as goiter only if the possibility of malignancy was excluded during the histopathological examination. The study includes 162 cores of thyroid samples from 70 female and 11 male patients acquired immediately after thyroidectomy. Clinical data, including age, sex, tumor stage T, and initial and histopathological diagnosis, are summarized in Table 1. Thyroid neoplasms were classified following the 2022 WHO Classification of Thyroid Neoplasms [97]. The protocol was approved by The Bioethics Committee of Nicolaus Copernicus University (KB470/2012).

### 4.2. Tissue Microarray (TMA) Preparation

Two independent pathologists verified and double-checked the TMA blocks. The archived FFPE tissue sections (donor blocks) were re-embedded in paraffin-wax tissue blocks, and H&E slides were made. Representative areas of at least 80% of tumor cells were marked on the hematoxylin-eosin-stained slides. Representative areas, 5 mm in diameter, were collected from the donor blocks and inserted into the recipient TMA block. For verification of the TMA blocks, hematoxylin-eosin slides were prepared.

### 4.3. Immunohistochemical Staining

Paraffin TMA blocks and archived formalin-fixed paraffin-embedded tissue sections were cut on a manual rotary microtome (AccuCut, Sakura, Torrance, CA, USA). Paraffin sections were prepared at a thickness of 3 µm and mounted onto extra adhesive slides (SuperFrostPlus, MenzelGlasser, Braunschweig, Germany). The immunohistochemical staining was performed according to previously described procedures and standardized using a series of positive and negative control reactions [98]. The positive control reaction was performed on a model tissue selected according to reference sources (The Human Protein Atlas http://www.proteinatlas.org, [99]) and the antibody datasheet. Breast samples for claudin-1 (Anti-Claudin 1 antibody [1C5-D9], ab115225, Abcam), and tonsil samples for claudin-4 (Anti-Claudin 4 antibody [ab53156], Abcam, Cambridge, UK). The negative control reactions were performed on additional analyzed tissue sections by substituting the primary antibody with 1% BSA (bovine serum albumin) diluted in PBS (phosphate-buffered saline).

Deparaffinization, rehydration, and antigen retrieval were performed by heating sections in Epitope Retrieval Solution high-pH at 95–98 °C for 20 min. (Dako, Agilent Technologies, Santa Clara, CA, USA) in PT-Link (Dako, Agilent Technologies). Subsequently, endogenous peroxidase activity was blocked with a 3% H_2_O_2_ solution for 15 minutes at room temperature (RT). The non-specific binding was blocked using a 5% solution of BSA for 15 min in RT. Incubation with the primary antibodies—claudin-1 (1/100, Anti-Claudin 1 antibody [1C5-D9], ab115225, Abcam), and claudin-4 (1/200, Anti-Claudin 4 antibody [ab53156], Abcam)—was performed. The antibody complex was detected using EnVisionFlex Anti-Mouse/Rabbit HRP-Labeled Polymer (Dako, Agilent Technologies) and localized using 3-3′ diaminobenzidine (DAB) as a chromogen. Finally, tissue sections were counterstained in hematoxylin, dehydrated, cleared in a series of xylenes, and coverslipped using a mounting medium (Dako, Agilent Technologies).

### 4.4. Image Acquisition and Analysis

**Figure 5:**
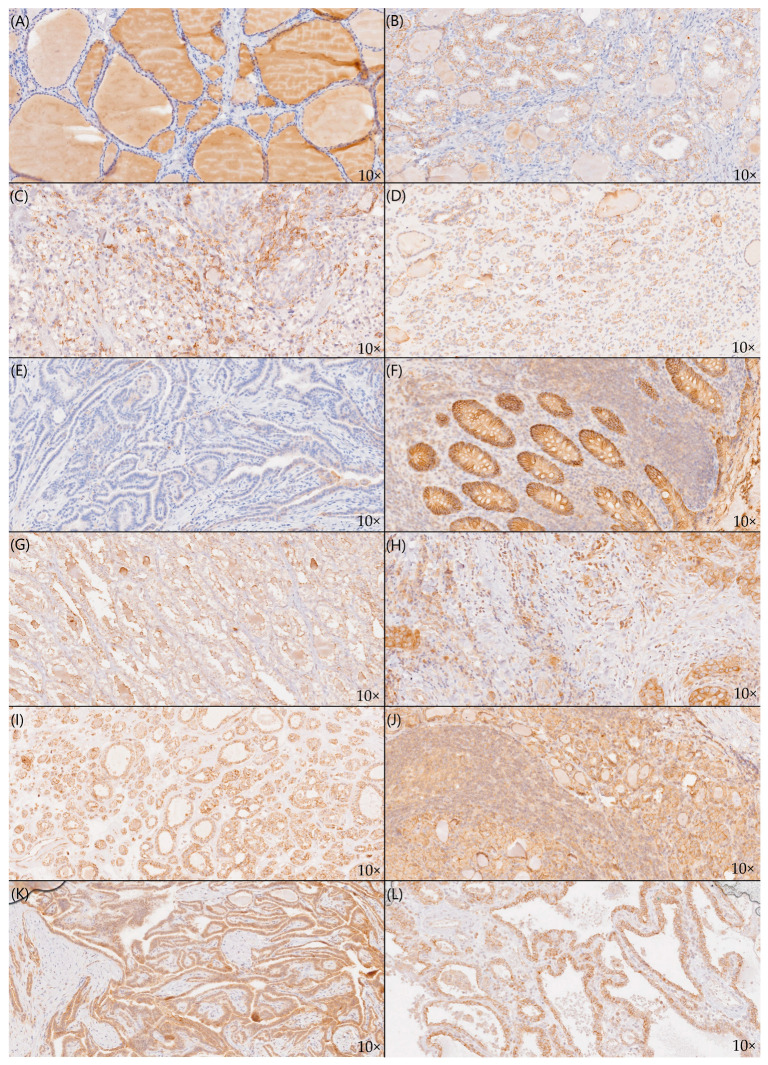
Cross-section staining patterns of (**A**) claudin-1 negative goiter; (**B**) claudin-1 negative anaplastic thyroid cancer; (**C**) claudin-1 positive thyroid adenoma; (**D**) claudin-1 negative thyroid adenoma; (**E**) claudin-1 negative papillary thyroid cancer; (**F**) claudin-4 positive control; (**G**) claudin-4 negative goiter; (**H**) claudin-4 negative anaplastic thyroid cancer; (**I**) claudin-4 negative thyroid adenoma; (**J**) claudin-4 positive thyroid adenoma; (**K**) claudin-4 positive papillary cancer; (**L**) claudin-4 positive papillary thyroid cancer.

Initially, the clinical data were blinded. The images were captured using an optical microscope at 10× magnification with a color video camera attached to a computer system. Two experienced pathologists selected the most representative regions for each sample and acquired images. The analysis used the ImageJ 1.53j version (NIH, Bethesda, MD, USA) (Java 1.8.0_172). All images were analyzed using the following protocol. First, the ImageJ “Threshold” function determined the area covered by analyzed tissues (goiter, adenoma, or cancer). Then, the “Color Deconvolution” function was used to separate hematoxylin and DAB staining. Using the “Threshold” function again, the area with positive DAB staining was determined. The expression was calculated as a percentage of the DAB-positive area in relation to all stained areas (tissue area). Cells were considered positive if the stainer area was at least 5% of the tissue area. Examples of cross-section staining in examined samples are shown in Figure 5.

### 4.5. In Silico Analysis

All data were obtained through the Human Pathology Atlas, a part of The Human Protein Atlas (www.proteinatlas.org). Transcriptome data were accessed using the XENA browser [100]. The TCGA cohort was accessed on 15 May 2024 and consisted of 501 patients diagnosed with papillary thyroid cancer [101,102]. The TCGA RNA-seq data were mapped using the Ensembl gene ID available from TCGA, and claudin-1 expression was quantified using the FPKMs (number Fragments Per Kilobase of exon per Million reads). The patients were classified into two expression groups based on the FPKM value. The best cutoffs were chosen using the Cutoff Finder web app [103]. The cutoff was set at 117.78 FPKM for claudin-1 and at 74.7 FPKM for claudin-4. Tumors with expression below the cutoff were assigned to the low expression group; otherwise, they were included in the high expression group. Differences in overall survival between groups were visualized using the Kaplan–Meyer estimator and calculated using the log-rank test. Results were considered statistically significant when *p* < 0.05.

### 4.6. Statistical Analysis

Statistical analysis was performed using Statistica version 13.3 (Statsoft) and Microsoft Excel 2019. The value of *p* < 0.05 was considered statistically significant. Continuous variables were tested for normality by the Kolmogorov–Smirnov test. The comparative studies used the *t*-test, the Mann–Whitney U, or the ANOVA Kruskal–Wallis tests.

## 5. Conclusions

Papillary cancers are characterized by high expression of claudin-1, and its low expression correlates with shorter 5-year survival of patients with papillary thyroid cancer (Figure 2 and Figure 3). Goiters, adenomas, and medullary and anaplastic cancers showed low or negative claudin-1 expression. Among the examined pathologies, only medullary and anaplastic thyroid cancer are characterized by negative claudin-4 staining, and there was no significant difference in claudin-4 staining between other thyroid pathologies (Table 2). While high claudin-1 expression is characteristic of papillary thyroid carcinoma, positive claudin-1 staining does not allow to distinguish benign pathologies, such as adenomas, from aggressive cancers, such as anaplastic thyroid cancer. It also does not discriminate between medullary thyroid cancer and anaplastic thyroid cancer. Clarifying the role of claudin-1 and claudin-4 in the pathogenesis of thyroid cancers may improve our understanding of tight junction proteins’ role in early metastasis.

To date, several preclinical studies exploring the role of claudin-1 targeted approaches in thyroid cancer have been reported. Downregulation of claudin-1 with various strategies, including PKC inhibition, *C. perfringens* enterotoxin, RET inhibition, or exogenic delivery of anti-cancer miRNA, may be beneficial in the case of PTC, FTC, and MTC. On the other hand, claudin-1 upregulation exhibits potential anti-metastatic and anti-cancer effects in ATC (FSTL1) and FTC (dexamethasone). Therefore, targeting claudin-1/4 may increase the efficiency of chemotherapy and immunotherapy. Claudin expression on the surface of cell membrane is a potential predictive biomarker that facilitates precise delivery of anti-can cancer agents. While anti-claudin treatment may suppress EMT and metastasis of tumors, several studies showed anti-EMT and anti-cancer activity of claudin-1 was demonstrated. It seems that claudin-1/4 activity is tumor-specific and may change during progression. However, to fully understand the role of both claudins in tumorigenesis and to develop effective targeted therapy, more in-depth studies are needed.

## Figures and Tables

**Table 1:** Basic characteristics of the research cohort.

Variables		*n* (%)
Sex	Female	70 (86.42%)
	Male	11 (13.58%)
Histological diagnosis	Goiter	62 (38.27%)
	Adenoma	52 (32.10%)
	Oncocytic adenoma	6 (3.7%)
	Papillary thyroid cancer	22 (13.58%)
	Medullary thyroid cancer	10 (6.17%)
	Anaplastic thyroid cancer	10 (6.17%)
Cancer stage	pT1	10 (23.18%)
	pT2	8 (19.05%)
	pT3	4 (9.52%)
	pT4	6 (14.29%)
	Unknown	14 (33.33%)

**Table 2:** Expression patterns of claudin-1 and claudin-4 in the research cohort.

Type of Tissue	Total (*n*)	Claudin-1			Claudin-4		
		Positive	Negative	*p*-Value	Positive	Negative	*p*-Value
Goiter	62	0	62	*p* < 0.0007 *^↟^	60	2	*p* = 0.816
Adenoma	58	18	40		42	16	
Cancer	42	16	26		22	20	
Papillary cancer	22	16	6	*p* < 0.0055 *^●^	20	2	*p* < 0.011 *^●^
Medullary cancer	10	0	10		0	10	
Anaplastic cancer	10	0	10		2	8	

*—statistical significance in expression between groups 1 and 2; ^●^—statistical significance in expression between groups 2 and 3; ^↟^—statistical significance in expression between groups 1 and 3.

**Table 3:** Basic characteristics of the TCGA cohort.

Clinical Data		*n* (%)	Claudin-1			Claudin-4		
			Low	High	*p*-Value	Low	High	*p*-Value
Age	≤45	235	106 (45.11%)	129 (54.89%)	*p* = 0.01	77 (32.77%)	158 (67.23%)	*p* = 0.008
	>45	266	149 (54.14%)	117 (45.86%)		59 (22.18%)	207 (77.82%)	
Sex	Female	366	180 (49.18%)	186 (50.82%)	*p* = 0.2	105 (28.69%)	261 (71.31%)	*p* = 0.2
	Male	135	75 (55.56%)	60 (44.44%)		31 (22.96%)	104 (77.04%)	
Race	White	332	159 (47.89%)	173 (52.11%)	*p* = 0.34	116 (34.94%)	216 (65.06%)	*p* = 0.196
	Asian	51	20 (39.22%)	31 (60.78%)		9 (17.65%)	42 (82.35%)	
	Black or African American	27	15 (55.56%)	12 (44.44%)		10 (37.04%)	17 (62.96%)	
Clinical stage	I	289	142 (49.13%)	147 (50.87%)	*p* = 0.001	89 (30.80%)	200 (69.20%)	*p* = 0.027
	II	52	36 (69.23%)	16 (30.77%)		6 (11.54%)	46 (88.46%)	
	III	111	53 (47.75%)	58 (52.25%)		31 (27.93%)	80 (72.07%)	
	IV	47	23 (48.94%)	24 (51.06%)		9 (9.15%)	38 (80.85%)	
Lymph node invasion	N0	223	131 (58.74%)	92 (41.26%)	*p* = 0.0001	46 (20.63%)	177 (79.37%)	*p* = 0.01
	N1	216	87 (40.29%)	129 (59.72%)		67 (31.02%)	149 (68.98%)	

**Table 4:** Univariate and multivariate analysis of prognostic factors in the PTC TCGA cohort.

Variable	Univariate Analysis			Multivariate Analysis		
	HR	95% CI	*p*-Value	HR	95% CI	*p*-Value
Age (≤45 vs. >45)	N/A	N/A	0.98	-	-	-
Sex (F vs. M)	0.51	0.18–1.4	0.19	-	-	-
Tumor stage (T1 vs. T2-T4)	0.47	0.11–2.06	0.097	-	-	-
Clinical stage (I vs II-IV)	0.29	0.1–0.84	0.023	0.3	0.1–0.86	0.025
Nodes (N0 vs. N1)	0.7	0.23–2.16	0.54	-	-	-
CLDN-1 (Low vs. High)	8.06	1.82–35.62	0.006	7.91	1.79–35	0.006
CLDN-4 (Low vs. High)	0.3	0.07–1.31	0.11	-	-	-

N/A—not available; HR refers to the relative risk calculated for the first variable.

## Data Availability

The data presented in this study are available on request from the corresponding author. The data are not publicly available due to ethical restrictions. Publicly available datasets were analyzed in this study. This data can be found on proteinatlas.com; references [98,99] (accessed on 8 July 2024).
